# Evaluating Web Retrieval–Assisted Large Language Models With and Without Whitelisting for Evidence-Based Neurology: Comparative Study

**DOI:** 10.2196/79379

**Published:** 2025-10-29

**Authors:** Lars Masanneck, Paula Zoe Epping, Sven G Meuth, Marc Pawlitzki

**Affiliations:** 1 Department of Neurology Medical Faculty and University Hospital Düsseldorf, Heinrich Heine University Düsseldorf Dusseldorf Germany

**Keywords:** neurology, large language models, artificial intelligence, evidence-based medicine, medical guidelines, information retrieval

## Abstract

**Background:**

Large language models (LLMs) coupled with real-time web retrieval are reshaping how clinicians and patients locate medical evidence, and as major search providers fuse LLMs into their interfaces, this hybrid approach might become the new “gateway” to the internet. However, open-web retrieval exposes models to nonprofessional sources, risking hallucinations and factual errors that might jeopardize evidence-based care.

**Objective:**

We aimed to quantify the impact of guideline-domain whitelisting on the answer quality of 3 publicly available Perplexity web-based retrieval-augmented generation (RAG) models and compare their performance using a purpose-built, biomedical literature RAG system (OpenEvidence).

**Methods:**

We applied a validated 130-item question set derived from the American Academy of Neurology (AAN) guidelines (65 factual and 65 case based). Perplexity Sonar, Sonar-Pro, and Sonar-Reasoning-Pro were each queried 4 times per question with open-web retrieval and again with retrieval restricted to aan.com and neurology.org (“whitelisted”). OpenEvidence was queried 4 times. Two neurologists, blinded to condition, scored each response (0=wrong, 1=inaccurate, and 2=correct); any disagreements that arose were resolved by a third neurologist. Ordinal logistic models were used to assess the influence of question type and source category (AAN or neurology vs nonprofessional) on accuracy.

**Results:**

From the 3640 LLM answers that were rated (interrater agreement: κ=0.86), correct-answer rates were as follows (open vs whitelisted, respectively): Sonar, 60% vs 78%, Sonar-Pro, 80% vs 88%, and Sonar-Reasoning-Pro, 81% vs 89%; for OpenEvidence, the correct-answer rate was 82%. A Friedman test on modal scores across the 7 configurations was significant (*χ*^2^_6_=73.7; *P*<.001). Whitelisting improved mean accuracy on the 0 to 2 scale by 0.23 for Sonar (95% CI 0.12-0.34), 0.08 for Sonar-Pro (95% CI 0.01-0.16), and 0.08 for Sonar-Reasoning-Pro (95% CI 0.02-0.13). Including ≥1 nonprofessional source halved the odds of a higher rating in Sonar (odds ratio [OR] 0.50, 95% CI 0.37-0.66; *P*<.001), whereas citing an AAN or neurology document doubled it (OR 2.18, 95% CI 1.64-2.89; *P*<.001). Furthermore, factual questions outperformed case vignettes across Perplexity models (ORs ranged from 1.95, 95% CI 1.28-2.98 [Sonar + whitelisting] to 4.28, 95% CI 2.59-7.09 [Sonar-Reasoning-Pro]; all *P*<.01) but not for OpenEvidence (OR 1.44, 95% CI 0.92-2.27; *P*=.11).

**Conclusions:**

Restricting retrieval to authoritative neurology domains yielded a clinically meaningful 8 to 18 percentage-point gain in correctness and halved output variability, upgrading a consumer search assistant to a decision-support-level tool that at least performed on par with a specialized literature engine. Lightweight source control is therefore a pragmatic safety lever for maintaining continuously updated, web-based RAG-augmented LLMs fit for evidence-based neurology.

## Introduction

Large language models (LLMs) are rapidly being explored in health care for abilities ranging from extraction, labeling, and interpretation of clinical data [1-4] to support for clinical decisions [5]. Despite these promising applications, LLMs often struggle with factual accuracy and reasoning and are known to “hallucinate” [6,7], which implies that they can produce fluent, seemingly authoritative responses that are entirely fabricated or factually incorrect.

Retrieval-augmented generation (RAG) has emerged as a promising strategy to mitigate hallucinations by constraining LLMs to use vetted reference material [8]. Rather than relying only on LLM output, a RAG-based system retrieves relevant information or documents (eg, clinical guidelines and PubMed articles) from a knowledge store (eg, a database or a web search) and requires the model to respond based on that content. By grounding outputs in a high-quality evidence-based context and optionally reporting the sources, systems such as the Almanac by Zakka et al [9] observed gains in factuality and safety over LLM or search products like ChatGPT or Bing when answering medical specialty questions. Large medical benchmarking efforts have further highlighted that RAG elevates smaller models to performance levels of larger, more resource-intensive LLMs [10,11]. In our previous work, we have highlighted the potential of web search–based RAG setups, with a general-purpose system by the commercial AI provider Perplexity [12] outperforming most other tested LLMs on a question set based on the guidelines for neurology according to the American Academy of Neurology (AAN) [13]. In this setup, the web-RAG Perplexity model was also the only one that did not hallucinate sources [13].

Although previous studies have already indicated that LLM-driven answering can surpass traditional web search in certain aspects of medical information accuracy and relevance [14], a combination of both could well shape access to medical information in the future. As dominant web search providers such as Google increasingly integrate LLMs into the search experience [15], and major LLM chat services like ChatGPT [16] and Claude [17] simultaneously move toward web-supported generation, this paradigm of combining LLM generation with web retrieval might become the new norm. While the combination of both technologies promises technical innovation and efficiency boosts [11,18], such a shift could have profound implications for how the public—including medical professionals and patients—access and evaluate online information. Furthermore, while a web-based system has the advantage of having continuous access to new information, it might also be overwhelmed by nonprofessional sources, motivating certain professional LLM system providers, such as OpenEvidence [19], to index only biomedical peer-reviewed literature.

**Figure 1:**
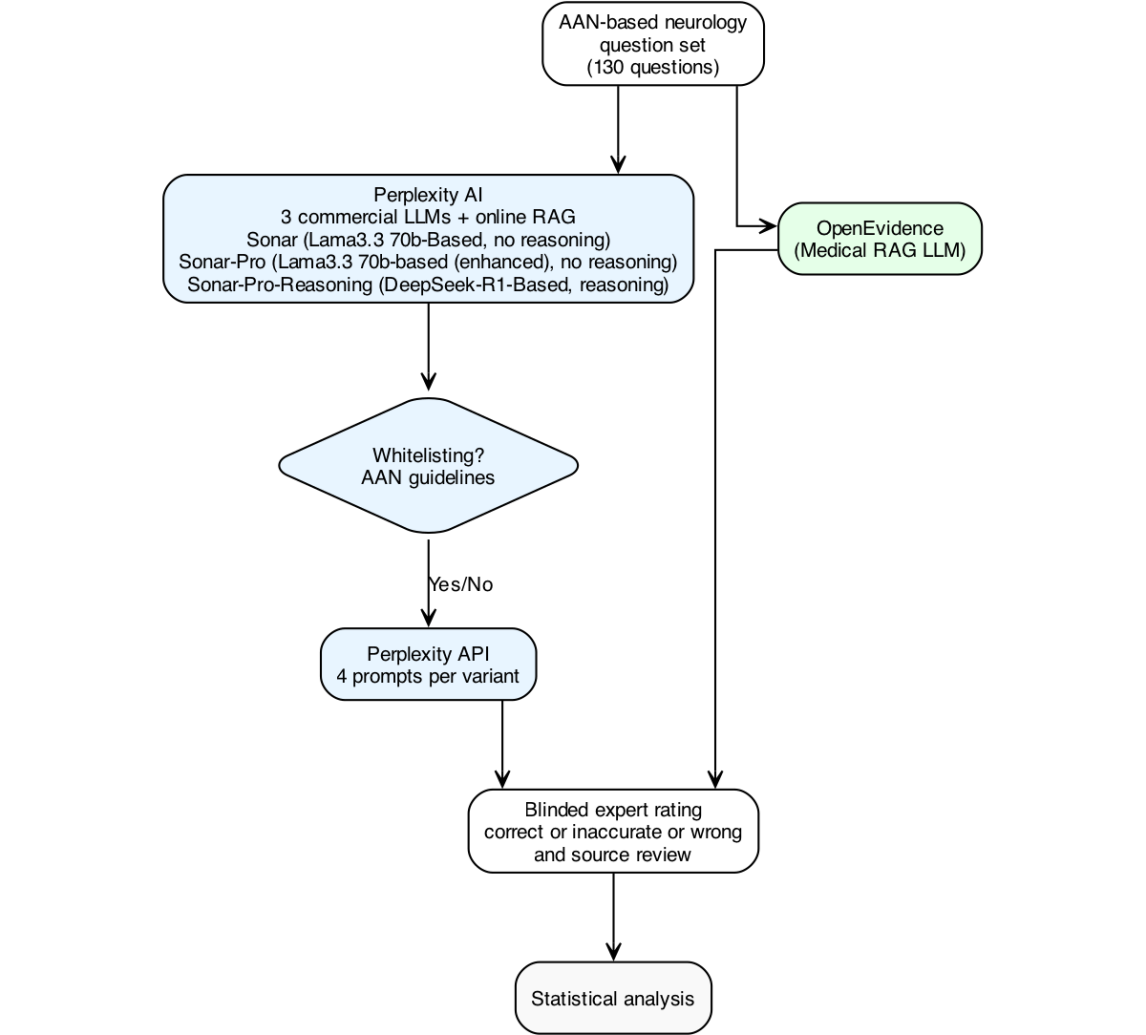
Experimental setup and study overview. Overview of the study setup for benchmarking clinical answer quality across commercial and medical domain web search–based large language model (LLM) products. A set of 130 American Academy of Neurology (AAN)-based neurology questions was asked using 3 Perplexity models—Sonar, Sonar-Pro, and Sonar-Reasoning-Pro—as well as OpenEvidence, a medical RAG system. Each Perplexity model was tested with and without whitelisting based on the AAN and neurology (AAN-related journals) domains. Outputs were rated by blinded experts on a 3-point scale (correct, inaccurate, and wrong) and assessed for type of source. API: application programming interface; RAG: retrieval-augmented generation.

Given these developments, we asked a pragmatic set of questions pertinent to clinicians already experimenting with “AI-powered search.” How well do widely available web-RAG assistants perform out of the box? By how much do they improve when their retrieval is restricted to authoritative guideline domains, and how do they compare with a purpose-built, literature-only tool? To address these questions, we evaluated Perplexity’s 3 public tiers on the previously published guideline 130-item AAN benchmark [13], first with the models’ default open-web retrieval and then with domain-level whitelisting to aan.com and neurology.org. To make a comparison, the same questions were posed to the professional medical OpenEvidence [19] available to health care professionals. See Figure 1 for a study outline.

## Methods

### Labeling Setup and Data Set

We evaluated the accuracy of answers returned by Perplexity’s 3 commercially available model tiers—Sonar, Sonar-Pro, and Sonar-Reasoning-Pro—queried with and without Perplexity’s native whitelisting option. Sonar, at the time of querying, was based on Llama 3.3 70B [20], with Sonar-Pro also based on the same model with additional capabilities (expanded context window of 200,000 tokens instead of 128,000, allowing it to process more input sources). Sonar-Reasoning-Pro was based on DeepSeek-R1 with weights publicly available [21]. Perplexity was selected as the application programming interface (API) for this experiment because of its broad functionality (including whitelisting) and its early leadership and visibility in web-grounded, RAG-assisted LLM answering.

To benchmark against a purpose-built medical system, we added OpenEvidence (no detailed technical information available), a RAG service that uses a proprietary index of biomedical literature and is available to health care professionals [19].

We used the previously published 130-item AAN benchmark with 65 clinical-case questions and 65 factual knowledge questions [13]. The underlying items were written de novo from AAN guidance and partially paraphrased; half are clinical vignettes that require applying multiple recommendations to a scenario rather than matching page wording. As all questions are based on respective AAN guidelines, which are published on the AAN website and also often published in the neurology family of journals, whitelisting options were set to restrict the search space to websites based on the aan.com and neurology.org domains. All Perplexity runs were executed on March 18, 2025; each model or whitelisting combination was prompted 4 independent times to capture answer variance (identical system or user prompts). OpenEvidence was queried 4 times per item between March 11 and 15, 2025 (however, identical answers were returned on repeat calls potentially owing to server-side caching).

### Labeling Process

Two physicians (LM and MP) with a background in neurology (clinical experience 5 years and 10 years, respectively) blindly scored every answer: 2=correct, 1=inaccurate, 0=wrong. A third senior neurologist (SGM; 21 years of experience) resolved any disagreements that arose. Raters were blinded to model and setup. As in a previous work [13], raters were instructed to classify an answer as “correct” if the recommendation itself was accurate even if minor errors in the reasoning process were present. Responses were labeled “inaccurate” if they were partially incomplete or contained even minor errors that could lead to misunderstanding of an otherwise generally correct answer. Responses were labeled “wrong” when the question was not answered properly or contained incorrect, very incomplete, or potentially medically harmful or misleading information. Different examples of these rating categories are shown in the referenced work [13] and in Table S1 in Multimedia Appendix 1.

A separate investigator (PZE) labeled every cited reference as professional (peer-reviewed journals, official guidelines, or professional society web pages) or nonprofessional (news sites, blogs, or generic health portals). Citations to aan.com or neurology.org were flagged as AAN related. None of the models showed any meaningful “source hallucination” [13]; therefore, a hallucination subanalysis was not conducted.

### Statistical Analysis

We addressed 2 prespecified questions. To ensure comparability with previous work [13], we performed a comparison using modal answers for questions for each condition and performed a Friedman test; significant results were followed up by post hoc Wilcoxon signed-rank pairwise comparisons with Holm multiple testing adjustments. For a more nuanced analysis of differences between nonwhitelisting and whitelisting models, we treated the ordinal rating scale as a quasi-interval and summarized the mean paired differences (Δ); additionally, we reported Cliff δ as an ordinal, distribution-free effect size. Owing to the ordinal underlying scale, whitelisting conditions were compared using Wilcoxon signed-rank tests and Holm corrections, and 95% CIs of mean changes were estimated using bias-corrected bootstrapping (5000 resamples).

We conducted two ordinal logistic regression analyses to test the influence of (1) the question type (knowledge vs case) and (2) retrieved source type on ordinal answer quality ratings. For each model category, we fitted an ordered-logit model. In the first analysis, we entered a single binary predictor containing the question type. In the second analysis, we instead entered 2 binary predictors—presence versus absence of an AAN or neurology journal citation and presence versus absence of a nonprofessional source (each with “absent” as the reference level). OpenEvidence was excluded from the second analysis as it never cites nonprofessional sources. We subjected the *P* values extracted from the Wald test again to Holm correction.

All analysis codes and underlying data, including model answers and ratings, can be accessed at the following GitHub repository [22].

### Ethical Considerations

No human subjects were involved in this research. As all analyses are based on a previously published data set without any actual patient data, in keeping with §15(1) of the Professional Code of Conduct (for physicians practicing in Germany) [23], ethics committee consultation is not required for conducting this study.

## Results

### Model Performance Across Configurations

The analysis generated 3640 answers to 130 questions from 3 Perplexity models (Sonar, Sonar-Pro, and Sonar-Reasoning-Pro), each with and without whitelisting, 4 replicates per prompt, plus 4 iterations of OpenEvidence per question (when trying to prompt multiple times, it showed no variation, presumably because of question caching). Interrater agreement between primary raters of answer quality was good at κ=0.86.

**Figure 2:**
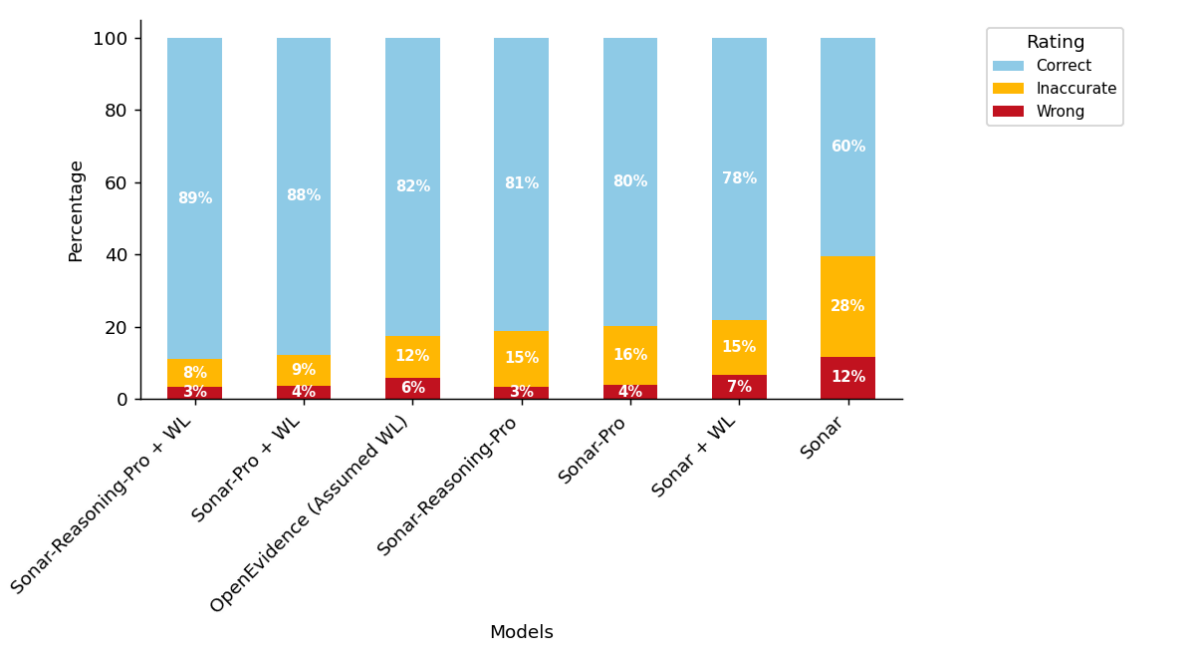
Overall response quality across models and configurations. Quality of generated answers under different system configurations, with and without the whitelisting (WL) of AAN domains (+WL). For OpenEvidence, the exact technical setup is not known, but some sort of whitelisting or indexing is assumed (assumed WL). Bars show the percentage of responses judged correct (light blue), inaccurate (orange), or wrong (red). AAN: American Academy of Neurology.

The basic model Sonar produced the lowest proportion of correct answers (314/520, 60.4%) and the highest rate of wrong answers (61/520, 11.7%). Answer quality improved with model capabilities to 79.8% (415/520) correct answers for Sonar-Pro and 81.2% (422/520) for Sonar-Reasoning-Pro. Whitelisting improved all these models to 78.1% (406/520; Sonar), 87.7% (456/520; Sonar-Pro), and 89% (463/520; Sonar-Reasoning-Pro). OpenEvidence, which as a product also works with indexed medical information, scored somewhere between these scores, at 82.5% (429/520) responses rated correct (Figure 2; Table S2 in Multimedia Appendix 1 for tabular representation). A Friedman test on modal ratings across all 7 model conditions was significant (*χ*^2^_6_=73.7; *P*<.001), with post hoc Holm-adjusted Wilcoxon tests showing all models, including whitelisting Sonar, significantly outperforming nonwhitelisting Sonar (all adjusted *P*<.01; see Table S3 and Figure S4 in Multimedia Appendix 1 for details). The top performer (Sonar-Reasoning-Pro + whitelisting) also exceeded Sonar + whitelisting (adjusted *P*<.001).

### Effects of Whitelisting on Answer Quality

**Figure 3:**
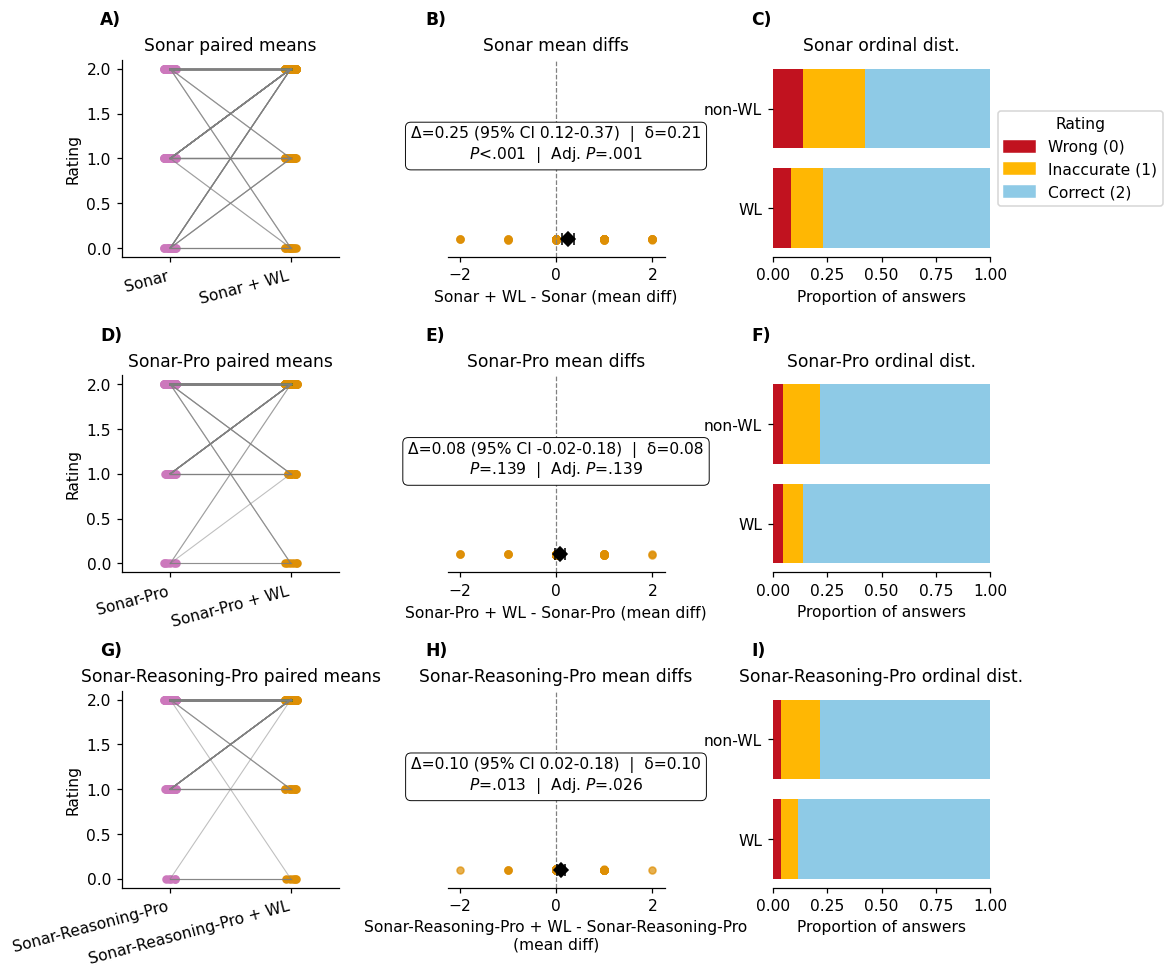
Impact of whitelisting (WL) on question‐level accuracy across Perplexity model setups. Panels A-C (Sonar), D-F (Sonar-Pro), and G-I (Sonar-Reasoning-Pro) each show, from left to right, paired means (A, D, and G): mean rating (0=wrong, 1=inaccurate, and 2=correct) for each question answered by the unaltered model (left) and the whitelisting-restricted version (right); gray lines connect the paired values. Mean differences (B, E, and H): black diamond with or without error bars mark the bias-corrected accelerated 95% bootstrap CI of the mean paired difference (Δ). The annotation box reports Δ (CI) | Cliff δ (ordinal effect size) on the first line, and the 2-sided Wilcoxon signed-rank *P* value together with its Holm-adjusted value on the second line. Positive values favor WL. Ordinal distribution (C, F, and I): stacked horizontal bars of the proportion of answers rated wrong (0; red), inaccurate (1; orange), or correct (2; blue) for non-WL versus WL. AAN: American Academy of Neurology; Adj.: adjusted; diff: differences; dist.: distributions.

Adding whitelisting systematically improved each Perplexity tier. Mean paired differences (Δ) on the 0 to 2 scale were 0.23 for Sonar (95% CI 0.12-0.34; Cliff δ=0.22, adjusted *P*<.001); 0.08 for Sonar-Pro (95% CI 0.01-0.16; Cliff δ=0.12, adjusted *P*=.02); and 0.08 for Sonar-Reasoning-Pro (95% CI 0.02-0.13; Cliff δ=0.20, adjusted *P*=.002; Figure 3). Of note, Cliff δ for all models also indicated a small effect.

### Influence of Question Type and Source Quality

When we analyzed the influence of the question type on answer quality, ordered-logit models showed higher accuracy on factual knowledge than case-based questions for every Perplexity configuration (odds ratio [OR] range from 1.95, 95% CI 1.28-2.98 (Sonar + whitelisting) to 4.28, 95% CI 2.59-7.09 (Sonar-Reasoning-Pro nonwhitelisting), all *P*<.01), whereas OpenEvidence displayed no such imbalance (OR 1.44, 95% CI 0.92-2.27, *P*=.11; see Figure S5 and Table S6 in Multimedia Appendix 1 for detailed statistics).

**Figure 4:**
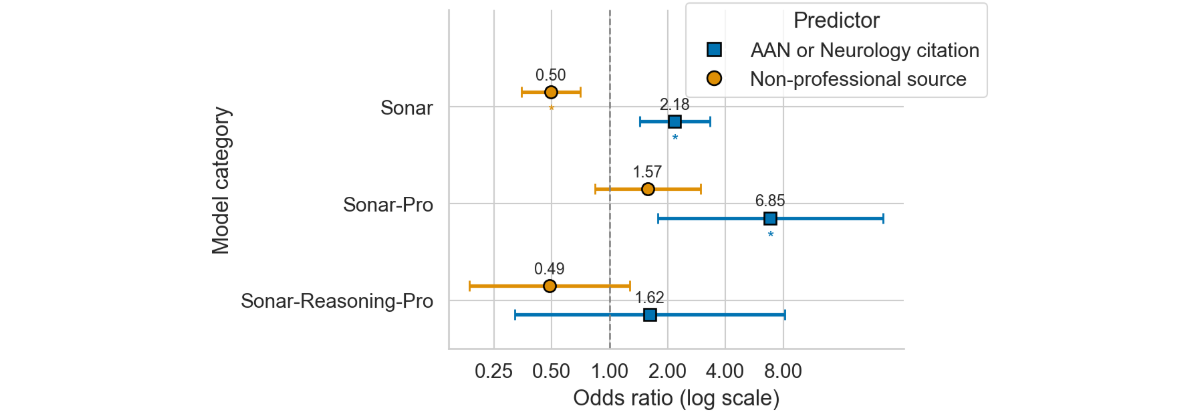
Forest plot of stratified ordinal logistic regression for predictors of question-level accuracy across Perplexity model categories. Each model (Sonar, Sonar-Pro, and Sonar-Reasoning-Pro) is listed on the y-axis and hosts 2 predictors, offset vertically: AAN Neurology Citation (square marker, blue graphic) and nonprofessional source (circle marker, orange graphic). Markers denote the estimated odds ratios (ORs) on a logarithmic x-axis, with horizontal whiskers showing 95% CIs. Numeric OR values are printed above each point; a dashed vertical line at OR=1 indicates the null effect. Significance after Holm correction is marked by an asterisk beneath the corresponding marker. AAN: American Academy of Neurology.

Similarly, the quality of the retrieved sources also had an influence on the response quality, especially for lower-tier models. For Sonar, retrieving ≥1 nonprofessional sources halved the odds of a higher rating (OR 0.50, adjusted *P*<.001), whereas inclusion of an AAN or neurology citation more than doubled it (OR 2.18, adjusted *P*<.001). The effect of source quality depreciated with model capabilities: only Sonar-Pro benefited significantly from guideline citations (OR 6.85, adjusted *P*=.01) while Sonar-Reasoning-Pro showed no significant source dependence (Figure 4; see Table S7 in Multimedia Appendix 1 for tabular representation).

Rating variability across batches of the same question varied from almost a third of questions (39/120, 32.5%) for the worst-performing model (Sonar; nonwhitelisting) to under a fifth for better performing models + whitelisting (eg, Sonar-Pro + whitelisting: 16/120, 13.3%; Figure S8 in Multimedia Appendix 1).

In other words, relatively, all but the weakest Perplexity configuration (Sonar; nonwhitelisting) surpassed the previously tested, supposedly larger online RAG Llama-3.1-405B model (67% correct) from the previous benchmark. The whitelisting setups of Sonar-Pro and Sonar-Reasoning-Pro performed even slightly better (1%-2%) than the previous best-performing model, which was a GPT-4o setup with direct access to only the relevant guidelines via RAG.

## Discussion

### Overview

In this comparative evaluation of commercially available online retrieval-augmented LLMs, answer accuracy scaled with both model capacity and—critically—domain-restricted retrieval. Activating Perplexity’s feature to restrict the search space to aan.com and neurology.org domains yielded absolute gains of 8 to 18 percentage points and halved the variance of stochastic outputs, most strikingly in the smaller Sonar tier. The best configuration achieved 89% guideline-consistent answers, outperforming a purpose-built, literature-only engine (OpenEvidence) and a previously tested specialized GPT-4o RAG system [13].

### Whitelisting as a Low‑Tech but High-Yield Intervention

By activating whitelisting, we converted a general‑purpose consumer tool into a near‑domain‑specific assistant without additional tuning, supporting recent evidence that retrieval quality—not just model scale—drives factual performance in medical RAG pipelines [9,10,13]. By cutting nonprofessional URLs out of the source pool, whitelisting also removes a major route for hallucinations and unsafe content, which pose a persistent threat to the factuality of LLMs.

Improvements were inversely proportional to baseline strength: the entry-level Sonar produced a wrong answer every eighth response, but whitelisting trimmed that error rate by one‑third. By contrast, Sonar‑Pro and Sonar‑Reasoning‑Pro were already strong; still, they gained approximately 8% in correct responses (although the “wrong” error rate was almost unaffected), underlining that source control remains worthwhile even for state‑of‑the‑art models. Ordinal-logit analysis confirmed the pattern: nonprofessional citations halved the odds of a correct answer in basic Sonar, whereas AAN or neurology journal links independently boosted accuracy in both Sonar and Sonar-Pro, with no significant effects in Sonar-Reasoning-Pro. While it is hard to extrapolate too much in this closed-model-setting, these findings suggest that fewer sources and less capable models benefit most from a cleaner evidence pool and from not having to judge a mixed-quality source. Across all tiers, factual knowledge items were easier than inferring case vignettes, a pattern consistent with earlier neurology [13] and multispecialty studies [24,25], and one that has likewise been observed among medical students in an earlier study [26]. This trend, interestingly, was not significant for the OpenEvidence comparison, which prompts further investigation into potential mediators and the underlying design of these products.

### Marginal Benefits of Explicit “Reasoning”

The DeepSeek-R1–based reasoning model was not markedly superior to the nonreasoning Sonar-Pro, with only minor, not clinically relevant improvements also observed in other traditional health care benchmarks such as the US medical examination, where DeepSeek-R1, for example, performed roughly on par with a nonreasoning GPT-4o model tested in another study [24,27]. As recently proposed open-ended and more gradual benchmarks such as OpenAI’s HealthBench show larger differences between both model types [28,29], this might also well be because many benchmarks—such as medical exams or the one used in this study—might be too simple for effective benchmarking.

### What Are Acceptable Thresholds?

Although the absence of statistically significant differences was noted, Perplexity’s higher tiers under whitelisting performed at least on par with OpenEvidence, a purpose-built, literature-only engine. This indicates that carefully scoped web retrieval can achieve performance comparable to curated medical indexes. Whether an 89% correctness rate in the best-performing system is sufficient for clinical decision support remains context-dependent and debatable. European regulators, for example, increasingly view medical‐domain LLMs as medical devices, triggering AI Act transparency and postmarket surveillance requirements [30,31]. The first European Conformité Européenne (CE)‑marked RAG-based LLM system (“Prof Valmed”) [32,33] secured its certification through transparent and traceable evidence links, which is precisely the mechanism that whitelisted, web search LLMs leverage. Nevertheless, there is no certainty on how certified medical products will compete with rapidly evolving, less-regulated industry alternatives, given the regulatory constraints that inherently limit adaptability [34]. Ultimately, decisions about system deployment are likely to hinge less on statistical significance and more on local clinical governance, effective postdeployment monitoring, clear accountability structures, and pragmatic user adoption. It also seems improbable that policymakers will significantly restrict medical web-based search applications powered by LLMs, considering they have not broadly intervened against general LLMs despite analogous concerns [35].

### Limitations

This study was limited to the single clinical domain of neurology. We further assume that our fixed whitelisting worked because neurology has well-defined, high-quality guidelines on certain domains. While we expect the general principle to work across all medical disciplines, the effect size of source curation might differ depending on guideline quality, guideline availability, and its algorithmic retrieval frequency. This highlights another limitation: the intransparency of the used commercial LLM (and search) systems, which might be updated silently after publication, thereby limiting reproducibility. One pragmatic way to mitigate this is to leverage open-weight analogs—open-source models with publicly available parameters—which would allow independent verification of results and scrutiny of model behavior [36]. Such open models can be continuously audited and fine-tuned, helping secure consistent performance even as proprietary systems evolve. Using open-source libraries [37], these can also be equipped with custom RAG setups, including grounding with search results. Importantly, alternative model setups might yield different absolute results, although we believe the observed trends would hold.

Furthermore, because the benchmark used here is derived from AAN guidance and several configurations restricted retrieval to AAN-related domains, domain-source coupling may have contributed to the observed gains. However, items were partially paraphrased, and the results did not produce a ceiling effect. Furthermore, a literature-only comparator (OpenEvidence, which likely contained AAN literature among many other) revealed a similar performance, suggesting that authoritative source control rather than site identity was the main driver. Nevertheless, this concept should be tested with other authoritative benchmark whitelisting combinations, for example, using AAN-based questions but whitelisting European neurology guidelines.

### Future Work: Potential of Request-Tailored Whitelisting

**Figure 5:**
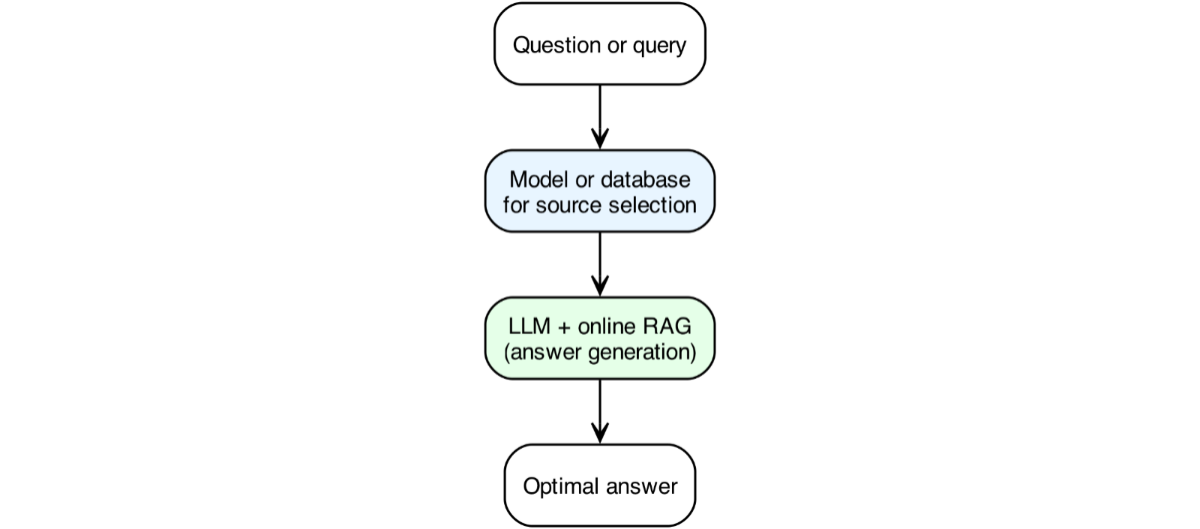
Conceptual domain-specific question-answer-pipeline balancing source control and recency. A proposed 2-stage architecture for future clinical question answering: incoming queries are first processed by a “Source Selector” module that dynamically retrieves, ranks, and whitelists evidence according to question type and medical specialty, which would ensure both domain relevance and up-to-date information. This could range from constituting a simple database to more complex models, potentially containing a large language model (LLM) + retrieval-augmented generation (RAG) system itself. The curated set of sources then feeds into the “whitelisting” part of an LLM + online RAG system to generate a structured, evidence-backed response.

For application across specialties or varying question types, one might need a more flexible approach, which is why we considered a 2-stage pipeline for future work:

1. A model to select sources—a lightweight agent (possibly database-linked) that sets the whitelisting based on query intent (eg, medical field, query type) and other factors such as user role.
2. LLM + RAG engine—operates only on the sources passed forward, dynamically delivering an evidence-backed answer (Figure 5).

Conceptually, this moves toward similar approaches with agentic LLM setups [38], which should be evaluated for trade-off between factuality, completeness, and recency in the future.

### Conclusions

Restricting retrieval improved correctness by 8 to 18 percentage points and reduced variability across all 3 web-RAG models, matching a dedicated professional literature-only engine. At the same time, the impact of source quality appears to be model-dependent. Taken together, retrieval control appears to be a low-friction safety lever for web-grounded LLMs with predefined authoritative sources. Further tests on generalizability and inclusion of more complex agentic systems are warranted.

## Appendix

Multimedia Appendix 1 Supplemental materials, including example answers, additional graphical representations, and more detailed tabular statistics.

## Abbreviations

AAN: American Academy of Neurology
LLM: large language model
OR: odds ratio
RAG: retrieval-augmented generation

## Data Availability

The data and the underlying code for this study can be found in the respective GitHub repository [22].

## References

[1] Azar WS, Junkin DM, Hesswani C, Koller CR, Parikh SH, Schuppe KC, Williams N, Nethala D, Mendhiratta N, Kenigsberg AP, Turkbey B, Merino MJ, Zaki G, Cortner J, Gurram S, Pinto PA (2025). LLM-mediated data extraction from patient records after radical prostatectomy. NEJM AI.

[2] Masanneck L, Schmidt L, Seifert A, Kölsche T, Huntemann N, Jansen R, Mehsin M, Bernhard M, Meuth SG, Böhm L, Pawlitzki M (2024). Triage performance across large language models, ChatGPT, and untrained doctors in emergency medicine: comparative study. J Med Internet Res.

[3] Ferber D, Wiest IC, Wölflein G, Ebert MP, Beutel G, Eckardt J, Truhn D, Springfeld C, Jäger D, Kather JN (2024). GPT-4 for information retrieval and comparison of medical oncology guidelines. NEJM AI.

[4] Kather JN, Ferber D, Wiest IC, Gilbert S, Truhn D (2024). Large language models could make natural language again the universal interface of healthcare. Nat Med.

[5] Gaber F, Shaik M, Allega F, Bilecz AJ, Busch F, Goon K, Franke V, Akalin A (2025). Evaluating large language model workflows in clinical decision support for triage and referral and diagnosis. NPJ Digit Med.

[6] Huang L, Yu W, Ma W, Zhong W, Feng Z, Wang H, Chen Q, Peng W, Feng X, Qin B, Liu T (2025). A survey on hallucination in large language models: principles, taxonomy, challenges, and open questions. ACM Trans Inf Syst.

[7] Xu Z, Jain S, Kankanhalli M Hallucination is inevitable: an innate limitation of large language models. arXiv.

[8] Lewis P, Perez E, Piktus A, Petroni F, Karpukhin V, Goyal N, Küttler H, Lewis M, Yih W, Rocktäschel T, Riedel S, Kiela D Retrieval-augmented generation for knowledge-intensive NLP task. arXiv.

[9] Zakka C, Shad R, Chaurasia A, Dalal AR, Kim JL, Moor M, Fong R, Phillips C, Alexander K, Ashley E, Boyd J, Boyd K, Hirsch K, Langlotz C, Lee R, Melia J, Nelson J, Sallam K, Tullis S, Vogelsong MA, Cunningham JP, Hiesinger W (2024). Almanac - retrieval-augmented language models for clinical medicine. NEJM AI.

[10] Xiong G, Jin Q, Lu Z, Zhang A (2024). Benchmarking retrieval-augmented generation for medicine. Proceedings of the 2024 Association for Computational Linguistics.

[11] Fernández-Pichel M, Pichel JC, Losada DE (2025). Evaluating search engines and large language models for answering health questions. NPJ Digit Med.

[12] API platform. Perplexity.

[13] Masanneck L, Meuth SG, Pawlitzki M (2025). Evaluating base and retrieval augmented LLMs with document or online support for evidence based neurology. NPJ Digit Med.

[14] Varghese J, Alen CM, Fujarski M, Schlake GS, Sucker J, Warnecke T, Thomas C (2021). Sensor validation and diagnostic potential of smartwatches in movement disorders. Sensors (Basel).

[15] Generative AI in search: let Google do the searching for you. Google.

[16] Introducing ChatGPT search. OpenAI.

[17] Claude can now search the web. Anthropic.

[18] Dewan M, Liu J, Gautam A, Shah C LLM-driven usefulness judgment for web search evaluation. arXiv.

[19] Home page. OpenEvidence.

[20] Meet new Sonar: a blazing fast model optimized for perplexity search. Perplexity.

[21] Today we're open-sourcing R1 1776, a version of the DeepSeek-R1 model that has been post-trained to provide unbiased, accurate, and factual information. Perplexity.

[22] MasanneckLab / Online-Search-Based-RAG.

[23] (Muster-)Berufsordnung für die in Deutschland tätigen Ärztinnen und Ärzte (MBO-Ä). Bundesärztekammer.

[24] Bicknell BT, Butler D, Whalen S, Ricks J, Dixon CJ, Clark AB, Spaedy O, Skelton A, Edupuganti N, Dzubinski L, Tate H, Dyess G, Lindeman B, Lehmann LS (2024). ChatGPT-4 omni performance in USMLE disciplines and clinical skills: comparative analysis. JMIR Med Educ.

[25] Singhal K, Azizi S, Tu T, Mahdavi SS, Wei J, Chung HW, Scales N, Tanwani A, Cole-Lewis H, Pfohl S, Payne P, Seneviratne M, Gamble P, Kelly C, Babiker A, Schärli N, Chowdhery A, Mansfield P, Demner-Fushman D, Agüera Y Arcas B, Webster D, Corrado GS, Matias Y, Chou K, Gottweis J, Tomasev N, Liu Y, Rajkomar A, Barral J, Semturs C, Karthikesalingam A, Natarajan V (2023). Large language models encode clinical knowledge. Nature.

[26] Haycocks NG, Hernandez-Moreno J, Bester JC, Hernandez R, Kalili R, Samrao D, Simanton E, Vida TA (2024). Assessing the difficulty and long-term retention of factual and conceptual knowledge through multiple-choice questions: a longitudinal study. Adv Med Educ Pract.

[27] Tordjman M, Liu Z, Yuce M, Fauveau V, Mei Y, Hadjadj J, Bolger I, Almansour H, Horst C, Parihar AS, Geahchan A, Meribout A, Yatim N, Ng N, Robson P, Zhou A, Lewis S, Huang M, Deyer T, Taouli B, Lee H, Fayad ZA, Mei X (2025). Comparative benchmarking of the DeepSeek large language model on medical tasks and clinical reasoning. Nat Med.

[28] Arora RK, Wei J, Hicks R, Bowman P, Quinonero-Candela J, Tsimpourlas F, Sharman M, Shah M, Vallone A, Beutel A, Heidecke J, Singhal K HealthBench: evaluating large language models towards improved human health. arXiv.

[29] Introducing HealthBench. OpenAI.

[30] Gilbert S (2024). The EU passes the AI Act and its implications for digital medicine are unclear. NPJ Digit Med.

[31] Freyer O, Wiest IC, Gilbert S (2025). Policing the boundary between responsible and irresponsible placing on the market of large language model health applications. Mayo Clin Proc Digit Health.

[32] We provide validated information for healthcare professionals. Prof. Valmed by Valmed Universe®.

[33] Instruction for use. Prof. Valmed by Valmed Universe®.

[34] Onitiu D, Wachter S, Mittelstadt B (2024). How AI challenges the medical device regulation: patient safety, benefits, and intended uses. J Law Biosci.

[35] Gilbert S, Harvey H, Melvin T, Vollebregt E, Wicks P (2023). Large language model AI chatbots require approval as medical devices. Nat Med.

[36] Hussain Z, Binz M, Mata R, Wulff DU (2024). A tutorial on open-source large language models for behavioral science. Behav Res Methods.

[37] Chase H LangChain. GitHub.

[38] Das R, Maheswari K, Siddiqui S, Arora N, Paul A, Nanshi J, Udbalkar V, Sarvade A, Chaturvedi H, Shvartsman T, Masih S, Thippeswamy R, Patil S, Nirni S, Garsson B, Bandyopadhyay S, Maulik U, Farooq M, Sengupta D Improved precision oncology question-answering using agentic LLM. medRxiv.

