# Supplementary material for: Evaluating Web Retrieval–Assisted Large Language Models With and Without Whitelisting for Evidence-Based Neurology: Comparative Study
Source: J Med Internet Res. 2025 Oct 29;27:e79379. doi: 10.2196/79379 (PMC12612646; doi:10.2196/79379)

Table S1: Exemplary answers with "wrong" or "inaccurate" rating

Question	Answer	Rating	Reason
According to current guidelines, should CT angiography, MRI angiography or conventional 4-vessel catheter angiography be used as ancillary tests to assist with the diagnosis of brain death?	Current guidelines from the American Academy of Neurology (AAN) do not specifically endorse the use of CT angiography, MRI angiography, or conventional 4-vessel catheter angiography as standard ancillary tests for brain death. However, these methods may be considered in specific contexts:<br>[…]<br>- **Ancillary Tests**: Guidelines primarily recommend tests like EEG, TCD, and cerebral scintigraphy for ancillary testing. These tests are to be used if clinical examinations are unreliable or apnea testing is inconclusive[1][3].	wrong	4-vessel catheter angiography may be used according to the current guideline. EEG is not recommended.
	(Hypothetical answer)<br>Current guidelines from the AAN mention 4-vessel catheter angiography as gold-standard for his task.<br>[…]<br>However, regarding CT angiography and MRI angiography there exist nor clear guidance […]	Inaccurate	This is ambiguous – at least according to current AAN guidelines both modalities are currently not recommended.
	1. Conventional 4-vessel catheter angiography is considered an acceptable ancillary test to aid in the diagnosis of brain death/death by neurologic criteria (BD/DNC).<br><br>2. CT angiography (CTA) is not recommended as an ancillary test for BD/DNC diagnosis according to the 2023 American Academy of Neurology (AAN) guidelines. The guidelines state that "Clinicians should not use CT angiography or magnetic resonance angiography as an ancillary test to aid in the diagnosis of BD/DNC."<br><br>3. MRI angiography is also not recommended as an ancillary test for BD/DNC diagnosis per the AAN guidelines.<br>[…]	Correct	Neither CT- nor MRI-angiography should be used. However, 4-vessel catheter angiography may be used according to the current guideline.

According to current guidelines, should EEGs, AEPs, or SEPs be used as ancillary tests to assist with the diagnosis of Brain Death?	According to current guidelines, EEG, auditory evoked potentials (AEPs), and somatosensory evoked potentials (SEPs) are not routinely required for brain death determination, but may be used as ancillary tests in certain situations:<br><br>The 2023 AAN/AAP/CNS/SCCM guideline on brain death/death by neurologic criteria (BD/DNC) determination states[1]:<br><br>- Ancillary testing is not mandatory but is recommended in certain circumstances, such as when components of the clinical examination or apnea testing cannot be safely performed or reliably evaluated.<br><br>- Acceptable ancillary tests include:<br>- Tests that assess cerebral blood flow (e.g. cerebral angiography, nuclear medicine perfusion scans)<br>- Tests that assess electrical activity of the brain (e.g. EEG)<br>[…]	Wrong	EEG should not be used in this case. Also AEPs and SEPs should not be used.
	(Hypothetical answer)<br>While EEG and AEP are not recommended by the AAN as ancillary tests for the diagnosis of brain death, I currently can't retrieve any information regarding SEPs.	Inaccurate	While none of this is wrong, an important piece of information – that SEPs are not recommended – is clearly lacking.
A 9-year-old girl has been treated for seizures for 4 years and her parents want to discuss the withdrawal of antiseizure medication with their neurologist. Is there evidence that an EEG can inform this decision?	*(Hypothetical answer)*<br>When discussing the withdrawal of antiseizure medication for a child, such as a 9-year-old girl who has been seizure-free for four years, EEG findings cannot provide any guidance as current guidelines state that EEG has no predictive power in children with seizures.	Wrong	This is simply going against the AAN guidelines – which recommend performing EEGs children.
	When discussing the withdrawal of antiseizure medication for a child, such as a 9-year-old girl who has been seizure-free for four years, EEG findings can provide some guidance, although the predictive value is not absolute. Here are key points to consider:	Inaccurate	While none of the here mentioned is really wrong, it does not tell us the specific recommendations by the AAN, which point toward:

	- **EEG Predictive Value**: Abnormal EEG results, especially those showing paroxysmal abnormalities, slowing, or spike and wave activities, have been associated with an increased risk of seizure relapse after medication withdrawal[1][5]. However, the predictive value of EEG should be evaluated within the context of other patient-specific factors[1].<br><br>- **Guidelines and Recommendations**: While specific recommendations from the American Academy of Neurology (AAN) on using EEGs for AED withdrawal are not detailed in the current search results, clinical practice generally involves a comprehensive assessment including EEG findings, seizure history, and patient characteristics[2].<br><br>- **Patient-Specific Factors**: Decisions to withdraw AEDs should be individualized, taking into account the patient's seizure type, duration of seizure freedom, age, and potential side effects of medication[2][4].<br><br>- **Duration of Seizure Freedom**: Most guidelines suggest at least two years of seizure freedom before considering AED withdrawal, though this can vary based on individual patient factors[2][4].<br>[…]		For children there is limited confidence that EEG abnormalities predict the recurrence of seizures in children. The guidelines recommend to order an EEG if ASM withdrawal is a possibility in the specific case. Withdrawal is only recommended as option if an EEG does not show an epileptoform activity.

Table S2: Distribution of Ratings by Model

Absolute counts and percentages of Correct, Inaccurate, and Wrong ratings for each model.

Model Category	Correct	Inaccurate	Wrong	Total	Correct (%)	Inaccurate (%)	Wrong (%)
Sonar-Reasoning-Pro + WL	463	39	18	520	89.0	7.5	3.5
Sonar-Pro + WL	456	45	19	520	87.7	8.7	3.7
OpenEvidence (Assumed WL)	429	60	31	520	82.5	11.5	6.0
Sonar-Reasoning-Pro	422	80	18	520	81.2	15.4	3.5
Sonar-Pro	415	85	20	520	79.8	16.3	3.8
Sonar + WL	406	79	35	520	78.1	15.2	6.7
Sonar	314	145	61	520	60.4	27.9	11.7

Table S3: Friedman Test & Post-hoc Wilcoxon Results

Friedman $\chi^2 = 73.728$, $P<.0001$

Model 1	Model 2	Original P-Value	P-Value Adjusted	Significantly Different
Sonar-Reasoning-Pro + WL	Sonar-Pro + WL	P=0.317	P>.99	No
Sonar-Reasoning-Pro + WL	OpenEvidence (Assumed WL)	P=0.064	P=0.706	No
Sonar-Reasoning-Pro + WL	Sonar-Pro	P=0.032	P=0.380	No
Sonar-Reasoning-Pro + WL	Sonar-Reasoning-Pro	P=0.013	P=0.169	No
Sonar-Reasoning-Pro + WL	Sonar + WL	P<.001	P=0.009	Yes
Sonar-Reasoning-Pro + WL	Sonar	P<.001	P<.001	Yes
Sonar-Pro + WL	OpenEvidence (Assumed WL)	P=0.266	P>.99	No
Sonar-Pro + WL	Sonar-Pro	P=0.139	P>.99	No
Sonar-Pro + WL	Sonar-Reasoning-Pro	P=0.147	P>.99	No
Sonar-Pro + WL	Sonar + WL	P=0.005	P=0.066	No
Sonar-Pro + WL	Sonar	P<.001	P<.001	Yes
OpenEvidence (Assumed WL)	Sonar-Pro	P=0.721	P>.99	No
OpenEvidence (Assumed WL)	Sonar-Reasoning-Pro	P=0.786	P>.99	No
OpenEvidence (Assumed WL)	Sonar + WL	P=0.186	P>.99	No
OpenEvidence (Assumed WL)	Sonar	P<.001	P<.001	Yes
Sonar-Pro	Sonar-Reasoning-Pro	P=0.862	P>.99	No
Sonar-Pro	Sonar + WL	P=0.346	P>.99	No
Sonar-Pro	Sonar	P<.001	P<.001	Yes
Sonar-Reasoning-Pro	Sonar + WL	P=0.266	P>.99	No
Sonar-Reasoning-Pro	Sonar	P<.001	P<.001	Yes
Sonar + WL	Sonar	P<.001	P=0.007	Yes

Figure S4: Heatmap of Post-Hoc Holm-Adjusted Wilcoxon Test Results for Language Model Ratings

Heatmap showing pairwise Holm-adjusted p-values for comparing language model (modal) ratings. The original Friedman test was statistically significant (Friedman $\chi^2 = 73.728$, $P<.0001$). Significance thresholds are indicated by a color gradient, with diagonal and upper triangle entries masked. See Table S3 for underlying values.

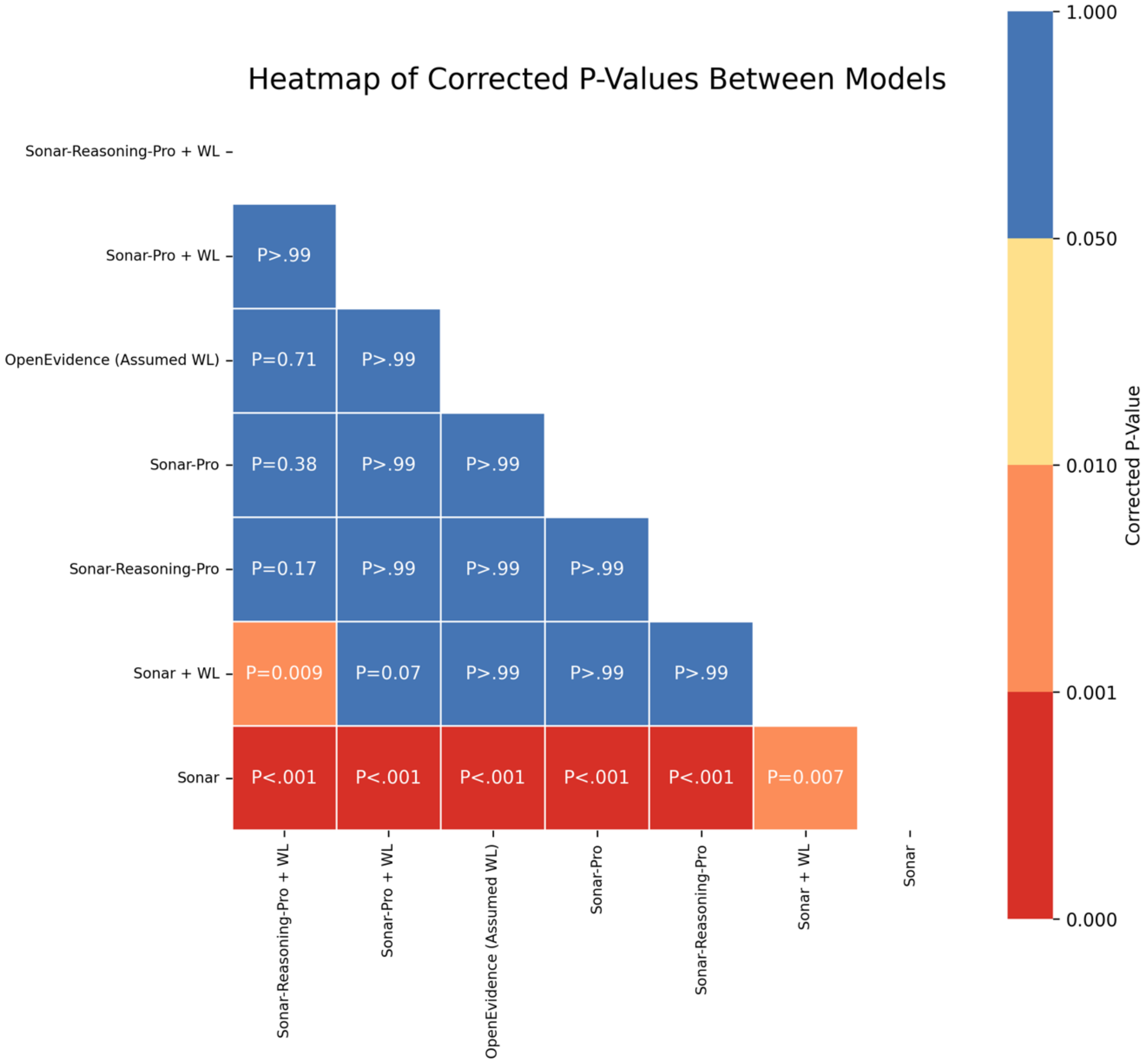

Figure S5: Quality and Source Referencing of LLM Responses for Cases and Knowledge Questions

(a) Stacked bar chart illustrating the percentage of answers in each rating category—“correct” (blue), “inaccurate” (yellow), and “wrong” (red) - when models responded to clinically oriented case-based questions.

(b) Corresponding stacked bar chart displaying the same rating categories for “knowledge” questions.

Due to rounding, summed percentages may not equal exactly 100%.

Results of an ordinal logistic regression comparing model performance model in both question types can be found in Table S5.

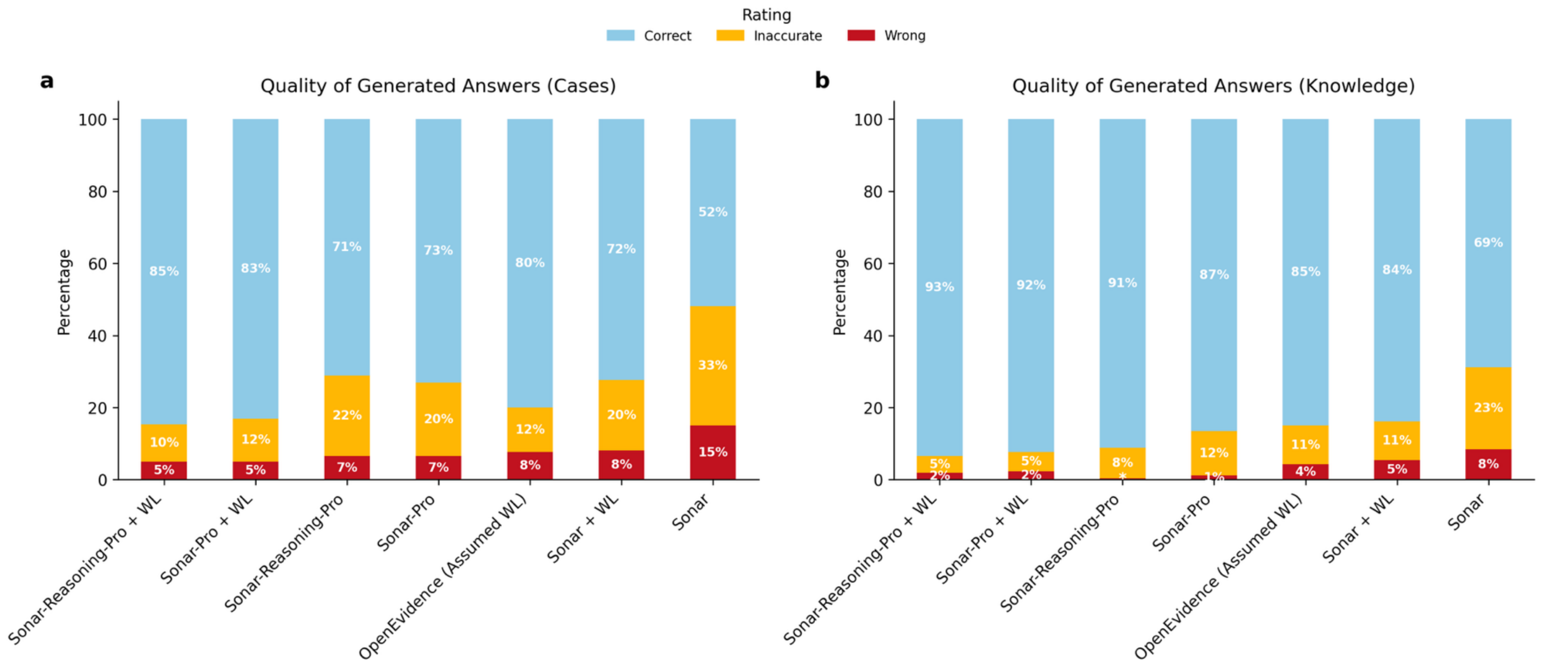

Table S6: Question Type Ordinal Logistic Regression Results by Model

Note: Odds Ratios represent the likelihood of higher performance ('Correct' > 'Inaccurate' > 'Wrong') for 'Knowledge' relative to 'Case'.

Model	P-value (Original)	P-value (Adjusted)	Significant?	Odds Ratio	95% CI Lower	95% CI Upper
OpenEvidence (Assumed WL)	P=0.114	P=0.114	No	1.443	0.9159	2.273
Sonar	P<.001	P<.001	Yes	2.025	1.428	2.871
Sonar + WL	P=0.002	P=0.007	Yes	1.951	1.275	2.984
Sonar-Pro	P<.001	P<.001	Yes	2.44	1.559	3.819
Sonar-Pro + WL	P=0.002	P=0.007	Yes	2.437	1.393	4.261
Sonar-Reasoning-Pro	P<.001	P<.001	Yes	4.283	2.586	7.093
Sonar-Reasoning-Pro + WL	P=0.002	P=0.007	Yes	2.601	1.434	4.718

Table S7: Source Type Ordinal Logistic Regression Results by Model

Note: Odds Ratios represent the likelihood of higher performance ('Correct' > 'Inaccurate' > 'Wrong') for each predictor, adjusted using Bonferroni correction.

Model	Feature	OR	P-value (Original)	P-value (Adjusted)	CI 2.5%	CI 97.5%
Sonar	Non-professional Source	0.496	P<.001	P<.001	0.3489	0.705
Sonar	AAN Neurology Citation	2.178	P<.001	P<.001	1.432	3.312
Sonar-Pro	Non-professional Source	1.574	P=0.16	P=0.16	0.8366	2.963
Sonar-Pro	AAN Neurology Citation	6.848	P=0.005	P=0.01	1.779	26.35
Sonar-Reasoning-Pro	Non-professional Source	0.4865	P=0.14	P=0.29	0.1856	1.275
Sonar-Reasoning-Pro	AAN Neurology Citation	1.616	P=0.56	P=0.56	0.3207	8.145

Figure S8: Variability in Question Ratings Across Batches by Sonar Model and Condition

Bar chart comparing the number of questions with differing ratings across various models. All setup of models and whitelisting condition answered to the same set of 130 guideline related questions four times. Variation decreased for all models with whitelisting. Better performing models also showed less variation.

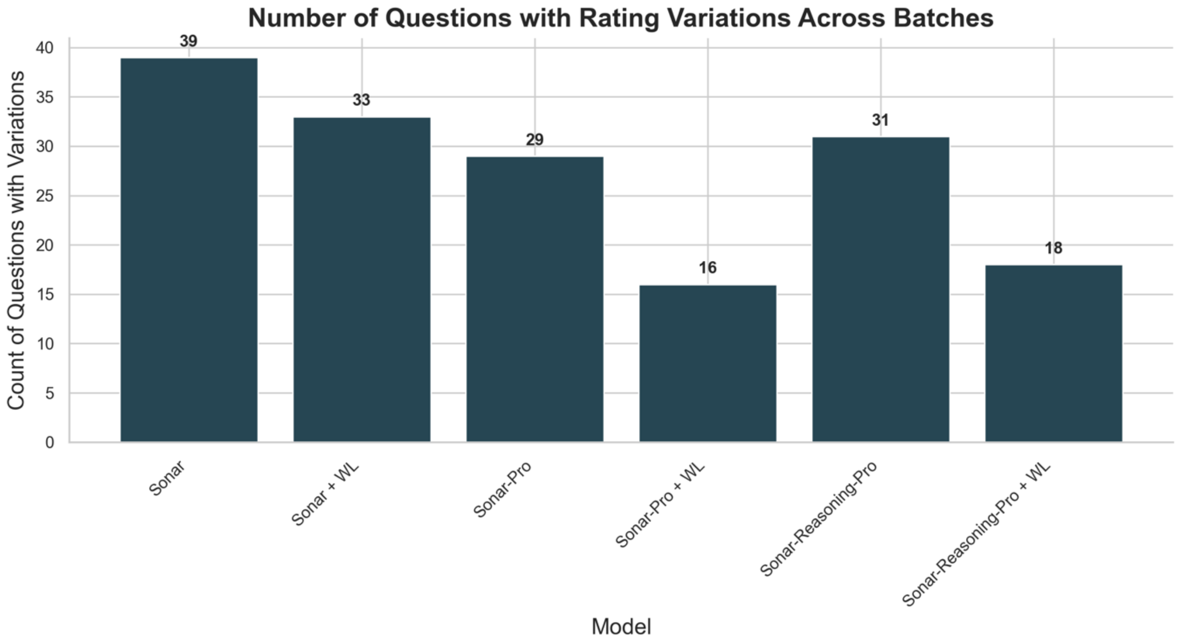

Supplement: Multimedia Appendix 1 [file jmir_v27i1e79379_app1.pdf]
